# Hyphal swelling induced in the phagosome of macrophages

**DOI:** 10.1016/j.funbio.2024.08.011

**Published:** 2024-11

**Authors:** María Fernanda Alonso, Judith M. Bain, Lars P. Erwig, Alistair J.P. Brown, Neil A.R. Gow

**Affiliations:** a Aberdeen Fungal Group, School of Medicine, Medical Sciences & Nutrition, Institute of Medical Sciences, University of Aberdeen, Foresterhill, Aberdeen, AB25 2ZD, UK; b Medical Research Council Centre for Medical Mycology, University of Exeter, Geoffrey Pope Building, Stocker Road, Exeter, EX4 4QD, UK

**Keywords:** Candida, Hyphae, Macrophage, Medical mycology

## Abstract

Macrophages play critical protective roles as sentinels of the innate immune system against fungal infection. It is therefore important to understand the dynamics of the interaction between these phagocytes and their fungal prey. We show here that many of the hyphal apices formed by *Candida albicans* within the macrophage ceased elongating, and apical and sub-apical hyphal compartments became swollen. Swollen hyphal cell compartments assimilated less Lysotracker-Red than non-swollen compartments, suggesting they had enhanced viability. Staining with florescent dyes suggested that there were higher levels of β-glucan and chitin in internalized fungal filaments compared to non-internalized hyphae, suggesting active cell wall remodelling within macrophages. These observations suggest that the stresses imposed by macrophages upon the fungus lead to changes in cell wall composition, inhibition of polarised growth and the induction of swelling in hyphal compartments, and that this can prevent or delay loss of viability of hyphal cells within the phagocyte.

## 1 Introduction

Fungal adaptation to changing environments often involves changes in cell morphology (Gow and Lenardon, 2022). *Candida albicans* is a morphologically plastic organism that can form a variety of cell types including yeast cells, hyphae, pseudohyphae, chlamydospores, opaque, gray, GUT (gastrointestinally induced transition), goliath cells and hyperpolarized buds (Gow and Yadav, 2017). The filamentous hyphal form has been linked to tissue invasion and pathogenesis (Kron and Gow, 1995; Mukaremera et al., 2017; Sudbery et al., 2004), although yeast, pseudohyphae and hyphal cells may all co-exist in infected host tissues. Hypha production is driven by a distinct transcriptional programme that regulates filament elongation and the co-expression of key virulence attributes such as the expression of critical hyphal cell wall proteins and the pore forming toxin candidalysin (Chen et al., 2020; Moyes et al., 2016; Wakade et al., 2023).

When yeast cells of *C. albicans* are phagocytosed by macrophages they quickly form filamentous cells (Lorenz et al., 2004). Hypha formation can lead to the disruption of the phagocytes by physical piercing of the phagocyte membrane, by activating candidalysin synthesis and inducing macrophage pyroptosis and the formation of extracellular traps, termed “ETosis” (Erwig and Gow, 2016; Lorenz et al., 2004; Olivier et al., 2022; Wellington et al., 2014). Hyphae that are too long to be completely phagocytosed can also be partially engulfed by macrophages which form a “frustrated phagosome” around the internalised part of the filament (Maxson et al., 2018). Macrophages can also fold longer *C*. *albicans* hyphae via an actin-dependent process (Bain et al., 2021). Yeast cells also occasionally promote their own expulsion from phagocytes via a process termed “vomocytosis” or “non-lytic expulsion” in which both the fungus and phagocyte survive (Bain et al., 2012).

In this study we report that the hyphae that are formed within the phagosome of macrophages can form swollen, bulbous apical and sub-apical compartments and that these swollen compartments retain their viability within the toxic environment of the phagolysosome. These observations add to other reports of morphological changes in *C. albicans* and suggest that swollen hyphae may represent a surviving and persisting sub-population of *C. albicans* cells within macrophages. Hyphal swelling may therefore be regarded as a mechanism of immune resistance.

## 2 Materials and methods

### 2.1 Fungal strains and growth conditions

*Candida* albicans SC5314 or a descendent (CA230) harbouring GFP under control of the *ACT1* promoter (Barelle et al., 2004) were maintained on frozen glycerol stocks until use. *C. albicans* strains were grown on YPD plates and incubated at 30 °C until colonies were formed. For macrophage interaction preparations, yeasts cells of *C. albicans* were cultured in YPD and incubated for 24 h at 30 °C, 200 rpm. In phagocytosis assays the multiplicity of infection (MOI) was set at 1:1 yeast cells: macrophages.

### 2.2 Thioglycollate-elicited peritoneal mouse macrophages

Peritoneal macrophages from C57BL/6 female mice were used from specific pathogen-free facilities at the University of Aberdeen. Macrophages were harvested from 10 to 14 week old mice and thioglycollate-elicited peritoneal macrophages were obtained from mice 3–4 days after intraperitoneal injection of 1 ml 3 % Brewer's thioglycollate broth (BD Bioscience). The peritoneal cavity was washed with 10 mL sterile, ice-cold 5 mM EDTA in phosphate-buffered saline (PBS) and washed twice by gentle centrifugation (400 g, 10 min) with RPMI 1640 Glutamax (Life Technologies) supplemented with 10 % (v/v) heat-inactivated foetal calf serum (Sigma), 200 U/mL penicillin/streptomycin (Sigma) and 10 mM HEPES (Life Technologies). In the phagocytosis assays, 1.5x10^5^ macrophages/well were used in 8-well μ-slides (ibiTreat surface, ibidi). The macrophages were first incubated in a CO_2_ incubator for 20–24 h at 37 °C, and then washed twice supplemented RPMI 1640 medium to remove non-adherent cells.

### 2.3 Live cell imaging

The conditions for phagocytosis were as described by (Lewis et al., 2013) at a MOI of 1:1. Macrophages were pre-stained with 50 mM LysoTracker Red (LTR) DND-99 (Invitrogen) after 1 h growth in supplemented RPMI 1640 medium. LysoTracker red DND-99 revealed the acidic phagolysosome compartments within macrophages (Lewis et al., 2013). An UltraVIEW VoX spinning-disk microscope (Nikon) with a ×40 or ×60 objectives in a 37 °C, 5 % CO_2_ environmental control chamber was used to carry out time lapse imaging. For 2D time lapse, images were captured every 2 min for a total of 4 h. For 3D recordings Z-stack images (0.5 μm step size) were captured every 10 min for a total of 8 h. In experiments using GFP expressing *C. albicans*, co-incubation was extended to 16 h and 3D images were acquired at this time point. Image analysis was performed using Volocity 6.3 software (Improvision, PerkinElmer)

### 2.4 Morphological analysis of internalised filaments

Maximum diameter (md) and diameter at septal junction (sd) were measured for individual compartments of *C. albicans* filaments growing within macrophages using Volocity 6.3 software (Improvision, PerkinElmer). Hyphae are defined as parallel sided tubular cells that contrast with pseudohyphae that have constrictions as septal junctions. Measurements were made between 210 and 240 min of image acquisition or immediately before compartment showed signals of cell death (assessed by permeability of LTR to fungal cell). Ratios of the two diameters were calculated. Hyphal compartments were classified as swollen if md/sd ≥ 2.0, intermediate if 1.5 ≤md/sd < 2 and non-swollen if md/sd < 1.5. These thresholds were conservatively defined and guided by visual observations to only consider compartments showing dramatic changes in normal, parallel-walled hyphal morphology. Percentages of filaments (mother cell and attached compartments) showing at least one swollen compartment or filaments showing no swollen compartments were determined. The percentage of swollen or non-swollen filaments with at least one compartment showing permeabilization to LTR were determined. The correlation of swelling and LTR permeabilization was assessed for individual swollen or non-swollen compartments. Diameters of non-internalized *C. albicans* cells within the same samples were measured as a control.

Volumes of swollen and non-swollen compartments of same length were determined after 16 h co incubation. Compartments of same size were identified and cropped using Volocity software. Volume was determined by object recognition using green fluorescence channel as a measure of GFP occupying the cytoplasm of *Candida* cells. Ratios of the two volumes (V_S_/V_NS_) were calculated for 10 swollen/non-swollen pairs.

### 2.5 Fungal cell wall staining and imaging

For 3D visualization of swollen compartments inside phagocytes, macrophage actin was stained with rhodamine phalloidin (Invitrogen) and fungal cell wall chitin was stained with Calcofluor White (CFW). After co-incubation, cells were fixed in 4 % paraformaldehyde for 45 min, permeabilized in 0.2 % Triton X 100 in PBS for 30 min and washed. Staining was performed sequentially using a final concentration of 5 μg/mL rhodamine-phalloidin for 20 min and 50 μg/mL CFW for 10 min. Z stack images (0.3 μm step size) were captured by spinning disk microscopy using a ×100 objective lens.

To analyse exposure of cell wall components in internalized swollen and non-swollen filaments, exposed mannan was stained with concanavalin A (ConA) conjugated to Alexa FluorTM 594 (Invitrogen), exposed chitin was stained with wheat germ agglutinin (WGA) conjugated to Alexa FluorTM 350 (Invitrogen) and exposed β-glucan was stained with Fc Dectin 1 (Graham et al., 2006) followed by goat F(ab')2 anti human IgG conjugated to Alexa FluorTM 488. After co incubation, macrophages were lysed with dH2O for 30 min and fungal cells were fixed in 4 % paraformaldehyde for 45 min and washed twice with PBS and once with staining buffer (1 × PBS, 1 % FCS, 0.5 mM EDTA). Staining was performed in two sequential steps. First, cells were incubated with 1.0 μg/mL Fc-Dectin-1 in staining buffer for 45 min on ice. Then cells were incubated with 50 μg/mL WGA, 25 μg/mL ConA and 1:200 Gt F(ab’) anti human IgG in staining buffer for 30 min on ice. Control cells stained only with the secondary antibody were included. All steps were followed by three washes with staining buffer. Z stack images (0.3 μm step size) were captured by spinning disk microscopy using a ×60 objective.

### 2.6 Statistical analyses

Mean values and standard deviations were calculated. Statistical significance was assessed by unpaired Student's t-test. P values of less than 0.05 was considered significant.

### 2.7 Ethics

The generation of peritoneal macrophages from mice was carried out in accordance with UK Home Office regulations defined within a Home Office project licence according to the terms and conditions of the United Kingdom Home Office licence 70/8073 for research on animals and the University of Aberdeen ethical committee. The use of animals in experiments and testing was regulated under the Animals (Scientific Procedures) Act 1986 (ASPA) and European Directive 2010/63/EU.

## 3 Results

### 3.1 Phagosomal environment alters hyphal morphology

**Fig. 1:**
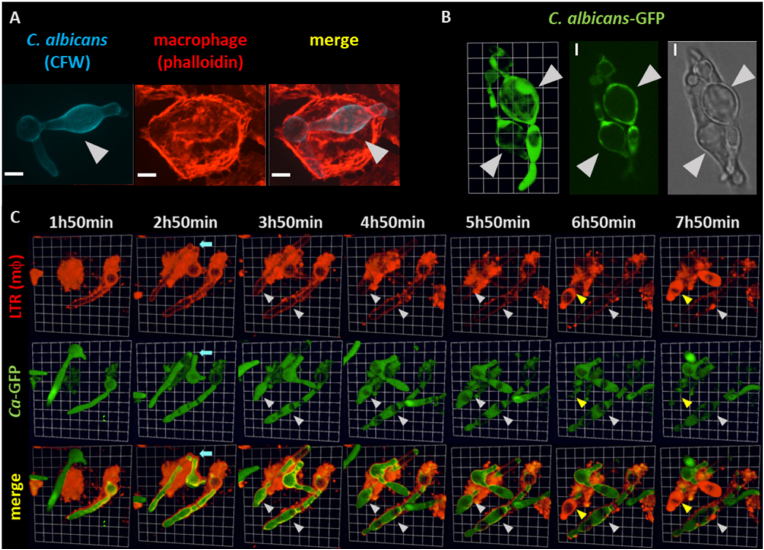
Phagosomal environment alters the morphology of internalized *C. albicans* filaments. A) Representative 3D image reconstruction (extended focus) showing aberrant filament morphology in *C. albicans* SC5314 internalized by a macrophage. Macrophage actin is stained with phalloidin (red) and fungal chitin is stained with CFW (blue). Swollen compartment is indicated with arrow. Cells were co-incubated for 6 h prior to imaging. Scale bar = 5 μm. B) Representative 3D image reconstruction (3D opacity) of two swollen *C. albicans* filaments (pACT1-GFP strain, green) inside a macrophage (left panel). Extreme morphological alterations were observed after 16 h co-incubation. Selected Z-stack from 3D reconstruction of GFP (middle panel) and DIC (right panel) channels show enlarged vacuoles in swollen compartments. Swollen compartments are indicated with arrows. 2D image scale = 3 μm; 3D image scale - 1 unit = 4 μm. C) Selected time point frames from 3D movie (Supplementary movie 1) in 3D opacity view mode showing interactions of macrophages with pACT1-GFP *C. albicans* (green). Macrophage acidic compartments are stained with LTR (red). Selected times are indicated in upper panel. Light blue arrows indicate macrophage fracture of internalized filament by septal junction. White arrowheads indicate swollen compartments. Yellow arrowheads indicate fungal compartment becoming permeable to LTR with the concomitant arrest in growth and loss of GFP signal, indicating cell death. Full movie of this sequence is provided as Supplementary movie 1. Scale 1 box unit = 3.5 μm.

**Fig. 2:**
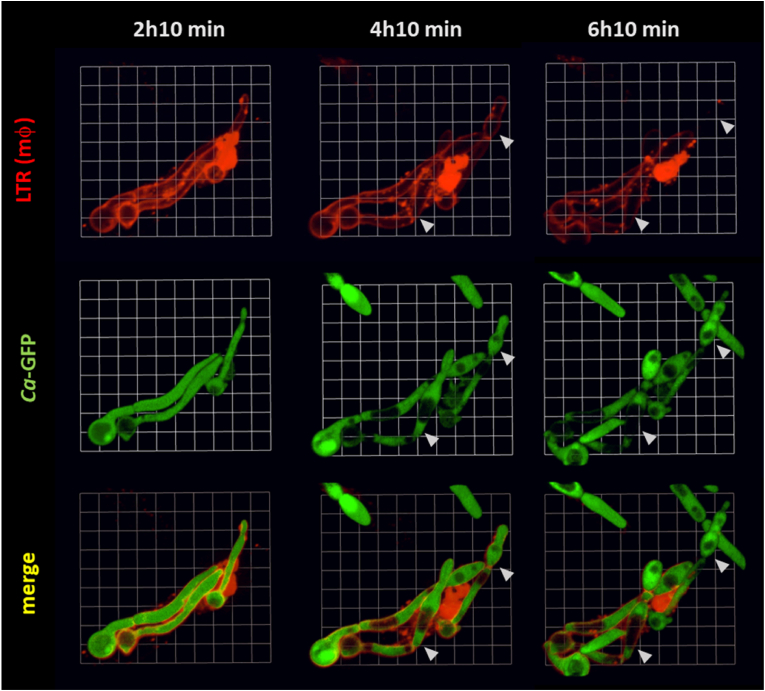
Elongation of swollen filaments inside the phagocyte resembled pseudohyphal growth. Selected time point frames from 3D movie (3D opacity) showing interactions of macrophages with pACT1-GFP *C. albicans* (green). Macrophage acidic compartments are stained with LTR (red). Selected times are indicated in upper panel. Sequence shows swelling of apical compartments of internalized fungal filaments. Subsequent elongation inside the phagocyte showed constrictions and ellipsoid compartments (indicated with white arrowhead). Scale 1 unit = 4.3 μm.

Imaging of *C. albicans* cells co-incubated with thioglycollate-elicited peritoneal macrophages revealed aberrant morphologies of internalized fungal filaments (Fig. 1A). Extreme examples of these morphologies were observed at prolonged co-incubation times (16 h) (Fig. 1B). Live cell imaging experiments revealed that *C. albicans* cells were primarily engulfed as yeasts or yeast cells that had formed short germ tubes. Initial growth of internalized cells exhibited normal unconstricted parallel-sided hyphal filaments. However, continued growth within phagosomal environment led to swelling of apical compartments (Fig. 1C & Supplementary movie 1). Subsequent filament elongation inside the phagocyte led to more swollen hyphal compartments superficially resembling pseudohyphae (Fig. 2). No differences in the incidence of hyphal swelling were observed within macrophages that had engulfed either one or multiple *C. albicans* cells.

**Fig. 3:**
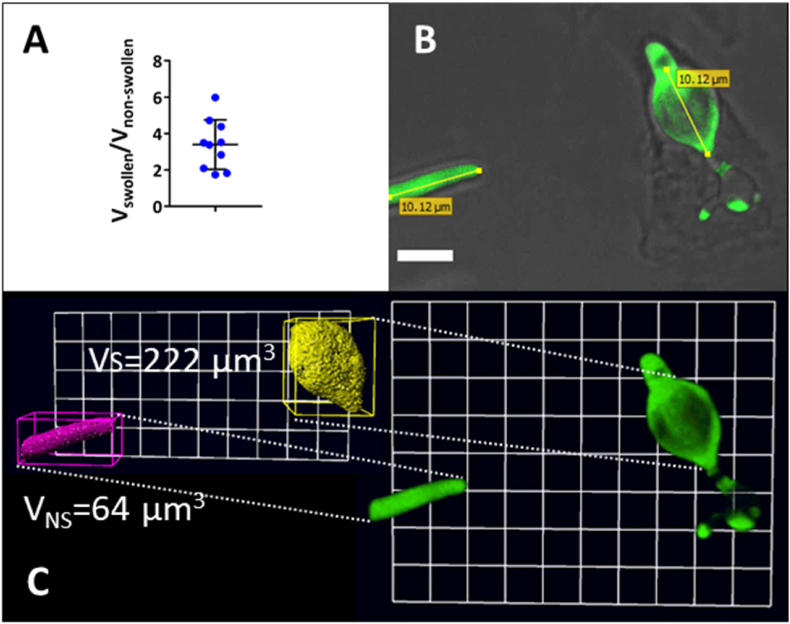
Volume of swollen *C. albicans* filaments can be at least 3 times larger than non-swollen compartments. Prolonged co-incubation (16 h) of macrophages with pACT1-GFP *C. albicans* resulted in extreme cases of filament swelling. A) Volumes of 10 swollen (V_S_) and non-swollen (V_NS_) compartments were measured using Volocity software. Error bars represent S.D's. B) Compartments of same length were selected (scale = 6 μm). C) Objects were automatically identified in the green channel (scale 1 unit = 3.6 μm). Swollen compartments were on average 3 times larger than non-swollen compartments (A).

At later time points (16 h), the volume of highly swollen compartments could be more than 3 times that of non-swollen compartments of the same length (mean of ratio of swollen: non-swollen compartment ± s.d, = 3.4 ± 1.4) (Fig. 3). The majority of this volume was occupied by an enlarged vacuolar compartment (Fig. 1B). No other changes, such as increased non-lytic expulsion of swollen cells, were noted.

### 3.2 Swollen hyphae are more resistant to phagosomal killing

**Fig. 4:**
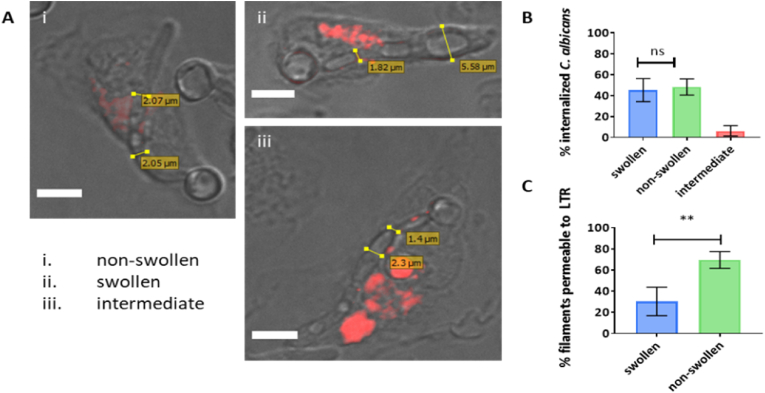
Morphological changes to apical compartments are frequent and more resistant to phagosomal killing. A) Representative DIC images of *C. albicans* SC5314 internalized by thioglycolate-elicited peritoneal macrophages (preloaded with LTR, red). Measurements of maximum diameter (md) and diameter at septal junction (sd) are shown for each cell. Cells were classified as non-swollen (i), swollen (ii) and intermediate (iii) according to md/sd ratio. Scale = 6 μm. B) Internalized filaments (mother cell and attached compartments) were defined as swollen if they exhibited at least one swollen compartment, non-swollen if they did not exhibit swollen compartments and intermediate otherwise. Percentages of internalized filaments falling into each category were calculated. Data are represented as mean ± SD of 4 biologically independent replicates. In total 45 cells were analysed per experiment. Statistical comparison between swollen and non-swollen groups was assessed by Student's t-test. C) Percentages of swollen or non-swollen filaments showing at least one compartment becoming permeable to LTR were calculated. Data are represented as mean ± SD of 4 biologically independent replicates. In total 45 cells were analysed per experiment. Statistical significance was assessed by Student's t-test. **P < 0.01.

True hyphae of *C. albicans* are characterised by having a constant diameter along their length. Divergence from a true hyphal morphology can be assessed by calculating the ratio between maximum diameter (md) and diameter at septal junction (sd). This ratio was measured for individual compartments of internalized *C. albicans* filaments and compartments were categorized as swollen, non-swollen or intermediate – for internalised cells only (Fig. 4A). Internalized filaments (mother cell and attached compartments) were defined as swollen if they exhibited at least one swollen compartment, non-swollen if they did not exhibit swollen compartments and otherwise intermediate. A comparable percentage of swollen (mean ± SD = 45 % ± 11 %) and non-swollen filaments (mean ± SD = 48 % ± 8 %) were identified after 4 h of co-incubation with macrophages (Fig. 4B). Intermediate filaments represented a small proportion (mean ± SD = 6 % ± 5 %) of internalized cells that may have been in transition to a swollen morphology. The latter were not used in further analyses. Swelling was never observed in non-internalized filaments, external to macrophages.

**Fig. 5:**
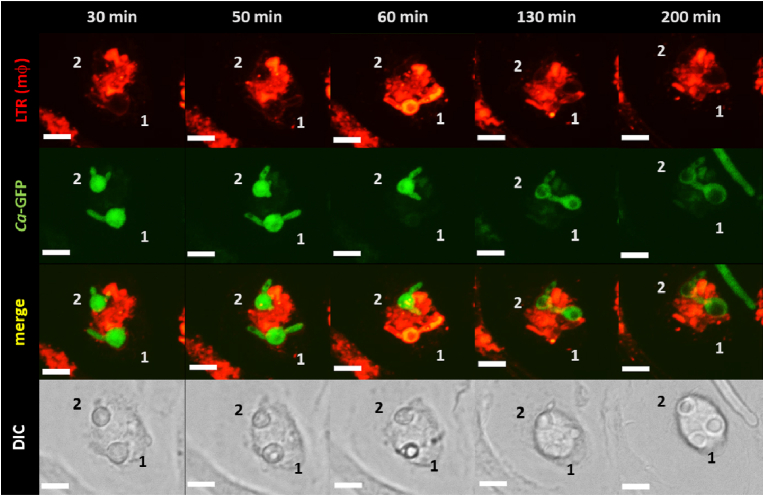
Fungal cell permeabilization to Lysotracker™ Red indicates cell death. Selected time point frames from 3D movie (extended focus) showing interactions of macrophages with pACT1-GFP *C. albicans* (green). Macrophage acidic compartments are stained with LTR (red). Selected times are indicated in upper panel. Sequence shows a fungal cell (1) internalized by a macrophage and showing initial elongation (30–50 min) within an acidified phagosomal compartment (indicated by LTR halo). Continued exposure to the phagocytic environment led to permeabilization of the fungal cell to LTR (60 min). This coincided with a complete arrest in fungal growth (60–200 min), change in the refractive index of the fungal cell as visualized by DIC and loss of GFP signal. The sequence also shows a fungal cell (2) internalized by a macrophage growing within a LTR positive compartment for the whole duration of the sequence. No signals of LTR permeabilization were observed. Scale = 5 μm.

To assess if swelling conferred an advantage or disadvantage to viability, death of fungal compartments was assessed by their permeabilization to LTR. Visual examination of the interactions between *C. albicans* cells and macrophages pre-loaded with LTR, showed that continued exposure to phagosomal content resulted in a percentage of internalized *C. albicans* cells becoming permeable to LTR. This coincided with cells exhibiting arrest in hyphal growth, and a change in the refractive index of the fungal cell as visualized by DIC, and the loss of GFP signal when using a GFP-transfected *C. albicans* strain (Fig. 5). These events, indicative in a change of viability, always occurred concomitantly. Thus, cell permeabilization to LTR was considered a good proxy for the evaluation of cell death.

A significantly higher percentage of non-swollen filaments (mean ± SD = 70 % ± 8 %) showed at least one compartment becoming permeable to LTR compared to swollen filaments (mean ± SD = 30 % ± 13 %) (Fig. 4C). A significant proportion of non-swollen internalized filaments that were permeable to LTR (mean ± SD = 37 % ± 6 %) showed limited elongation within the phagocyte and indications of whole filament death (e.g. death of the mother cell compartment). A direct comparison between swollen and non-swollen compartments of the same filament efficiently elongating within macrophages, revealed reduced cell death for swollen compartments (mean ± SD = 15.33 % ± 10 %) compared to non-swollen compartments (mean ± SD = 38 % ± 4 %) (n = 3, p < 0.05).

### 3.3 Alterations to the cell wall of internalized filaments

**Fig. 6:**
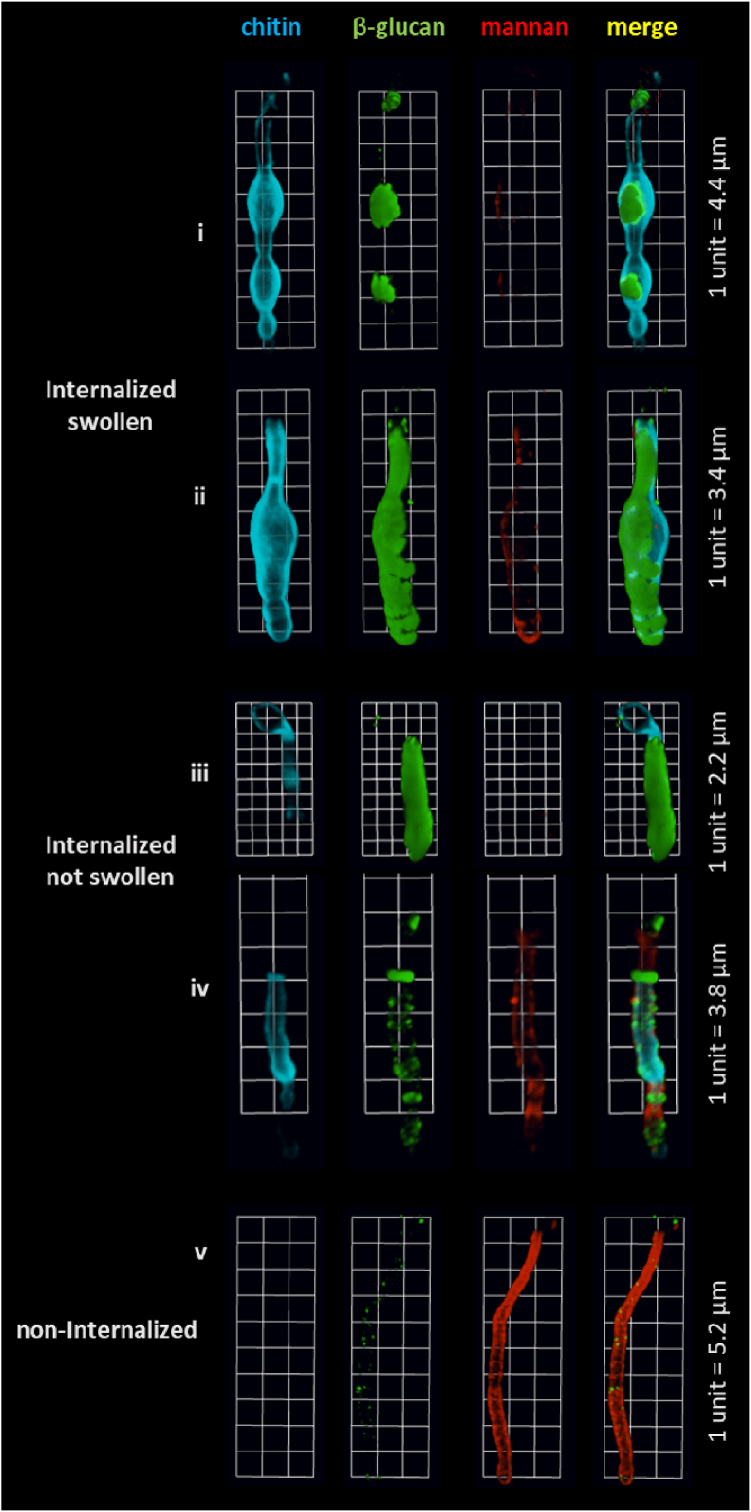
Internalized filaments show lower levels of mannan and increased exposure of β-glucan and chitin. Exposure of cell wall mannan (ConA, red), β-glucan (Fc-Dectin-1, green) and chitin (WGA, blue) was assessed for internalized swollen and non-swollen *C. albicans* filaments after 4 h co-incubation with macrophages. Representative 3D image reconstructions (3D opacity) of cell wall staining patterns of internalized swollen (and ii), internalized non-swollen (iii and iv) and non-internalized filaments (v). Scales are indicated in figure for each image.

Morphological changes are often associated with changes in cell wall architecture (Gow and Lenardon, 2022). Therefore, mannan, β-glucan and chitin exposure in swollen and non-swollen internalized filaments were compared. Non-internalized cells were used as a control to normalise any cell wall changes. Internalised hyphae were significantly different from non-internalised hyphae with regard to their mannan, β-glucan and chitin exposure (Fig. 6). Lower levels of mannan and higher levels of β-glucan and chitin were observed in swollen and non-swollen internalized filaments compared to non-internalized hyphae (Fig. 6).

## 4 Discussion

The phagosome is a hostile environment that induces adaptive changes in fungal cells as they attempt to resist killing. Limited access to nutrients, low pH, accumulation of reactive oxygen and nitrogen species and hydrolases contribute to the toxic environment of the macrophage phagolysosome (Erwig and Gow, 2016). In response, *C. albicans* activates several signalling pathways that control a series of transcription factors that counter and repair oxidative and nitrosative damage, and stresses imposed by cationic, osmotic, acid, and other stressors (Chiranand et al., 2008; da Silva Dantas et al., 2010,2006; Enjalbert et al., 2006; Kaloriti et al., 2014; Navarro-García et al., 1995; Patterson et al., 2013; Pradhan et al., 2018; Zhang et al., 2000). *C. albicans* can also inhibit or delay phagolysosomal maturation by interfering with RAB GTPase localization to macrophage phagosomes (Okai et al., 2015), induce its own expulsion or prevent phagosome acidification, express protective superoxide dismutases and the Yhb1, flavohaemoprotein, and induce macrophage pyroptosis leading to its expulsion (Erwig and Gow, 2016). In this work we demonstrate that the formation of swollen hyphal compartments may also contribute to this raft of protective measures deployed by *C. albicans* within the macrophage.

Swelling of internalized filaments occurred at high frequency in the experimental set-up used in this study. These morphological alterations were most noticeable after co-incubation times of >3.5–4 h, which are longer than those used regularly in phagocytosis assays with *C. albicans* (<3). The macrophage model used (*i.e.* thioglycolate-elicited peritoneal macrophages) and other experimental variables (e.g. low MOI) may also explain the enhanced number of swelling events identified in this study compared to previous reports.

Live cell imaging was used to follow the dynamic morphological changes of elongating *C. albicans* filaments within macrophages over time. Fungal cells initiated polarized growth, but prolonged exposure to the phagosomal environment disrupted apical growth at the expense of isotropically expanding cells. However, in some specimens, a subset of cells resumed polarized growth and the resultant filaments resembled pseudohyphae.

The molecular mechanisms underlying these morphological changes are unknown. However, similar morphotypes have been observed in *C. albicans* (Akashi et al., 1994; Crampin et al., 2005; Hazan and Liu, 2002) and in other filamentous fungi (Gupta and Heath, 1997; Torralba et al., 1998) treated with actin-disrupting drugs such as cytochalasin A. Disruption of actin cables has been associated with disassembly of the Spitzenkörper (Crampin et al., 2005) and random dispersion of secretory vesicles, resulting in isotropic growth.

The environmental cues leading to filament swelling within the phagosome remain to be elucidated. Reports of pH regulated morphogenesis of *C. albicans* noted a “tube that swells into an extremely elongated bud” when the external pH of the medium was lowered from 6.7 to below 6.0 (Buffo et al., 1984). During the process of phagosome maturation, the phagocytic compartment becomes increasingly acidic, with the pH reaching as low as 4.5 (Flannagan et al., 2012). Previous studies have described the ability of *C. albicans* to release NH_3_ that can neutralize the phagosomal environment and initiate hyphal growth (Danhof and Lorenz, 2015; Vylkova and Lorenz, 2014; Zhao et al., 2022). However, the membrane permeability of NH_3_ has been shown to be too high to allow neutralisation via the transport of ammonium (Westman et al., 2018; Freeman et al., 2023). However, these observations were made for early time points and did not explore the impact of macrophages lowering phagosomal pH with prolonged co-incubation times (Freeman et al., 2023; Westman et al., 2018). It is also known that the immunomodulatory phosphoprotein PTMA derived from macrophage lysates strongly induces *C. albicans* filamentation (Case et al., 2021).

It is also possible that phagosome hydrolases could degrade structural components of the wall such as chitin and β-1,3 glucan resulting in osmotically driven swelling. Early studies of osmotically induced hyphal growth arrest noted that this resulted in apical swelling (Robertson, 1958). Cell wall staining of internalized filaments revealed lower mannan levels and higher β-glucan and chitin exposure in swollen and non-swollen compartments compared to non-internalized filaments. Higher exposure of internal cell wall components in ingested fungi (Wagener et al., 2017) suggests a degree of cell wall degradation. The degradative properties of the phagosomes were also evidenced by their ability to fracture internalized filaments at septal junctions to expose β-glucan and chitin, and the ability of macrophages to fold longer hyphae (Bain et al., 2021). However, no major differences in cell wall staining were observed between internalized swollen and non-swollen compartments. Other studies have also suggested that acidic environments enhance the exposure of β-glucan and chitin (Sherrington et al., 2017).

Importantly, fewer swollen compartments showed signals of cell death compared to non-swollen compartments. Swelling may therefore be interpreted as a possible adaptation that promotes survival within the phagolysosome of macrophages.

## 5 Conclusion

Here we report a series of novel events following engulfment *of C. albicans* – the arrest of apical growth and the swelling of hyphae within the phagolysomes – that were associated with changes in the cell walls of the internalised filaments. Unexpectedly, swollen compartments with nearly triple the volume of normal parallel sided hyphae, resisted the uptake of LTR (a marker of reduced cell viability) indicating that hypha swelling may contribute to the ability of *C. albicans* to resist macrophage mediated killing.

## CRediT authorship contribution statement

**María Fernanda Alonso:** Writing – review & editing, Writing – original draft, Visualization, Validation, Methodology, Investigation, Formal analysis, Data curation, Conceptualization. **Judith M. Bain:** Writing – review & editing, Methodology, Investigation. **Lars P. Erwig:** Writing – review & editing, Conceptualization. **Alistair J.P. Brown:** Writing – review & editing, Writing – original draft, Funding acquisition, Conceptualization. **Neil A.R. Gow:** Writing – review & editing, Writing – original draft, Visualization, Validation, Resources, Project administration, Funding acquisition, Conceptualization.

## Declaration of competing interest

The authors declare no financial interests/personal relationships which may be considered as potential competing interests.
